# Application of ATR-FTIR for Green Arabica Bean Shelf-Life Determination in Accelerated Storage

**DOI:** 10.3390/foods13152331

**Published:** 2024-07-24

**Authors:** Sai Aung Moon, Sirirung Wongsakul, Hiroaki Kitazawa, Sila Kittiwachana, Rattapon Saengrayap

**Affiliations:** 1 School of Agro-Industry, Mae Fah Luang University, Chiang Rai 57100, Thailand; 6471401001@lamduan.mfu.ac.th (S.A.M.); sirirung@mfu.ac.th (S.W.); 2 Coffee Quality Research Group, Mae Fah Luang University, Chiang Rai 57100, Thailand; 3 Integrated AriTech Ecosystems Research Group, Mae Fah Luang University, Chiang Rai 57100, Thailand; 4 Department of Food and Nutrition, Faculty of Human Sciences and Design, Japan Women’s University, 2-8-1 Mejirodai, Bunkyo-ku, Tokyo 112-8681, Japan; kitazawah@fc.jwu.ac.jp; 5 Department of Chemistry, Faculty of Science, Chiang Mai University, Chiang Mai 50200, Thailand; sila.k@cmu.ac.th

**Keywords:** infrared spectroscopy, multivariate analysis, oxidation, rancidity

## Abstract

Coffee bean oxidation is associated with enzymatic and non-enzymatic browning, the degradation of desirable aromatic compounds, the development of undesirable flavors, increased susceptibility to microbial spoilage, and volatile compound losses. This study investigated natural dry process (DP) and honey process (HP) green coffee beans stored in GrainPro^^®^^ bags for 0, 5, 10, and 20 days under accelerated storage conditions at 30 °C, 40 °C, and 50 °C with relative humidity of 50%. A kinetic model was used to estimate the shelf life of the green coffee beans. DP recorded durability of 45.67, 29.9, and 24.92 days at 30 °C, 40 °C, and 50 °C, respectively, with HP 60.34, 38.07, and 19.22 days. Partial least squares (PLS) analysis was performed to build the models in order to predict the shelf life of coffee based on peroxide (PV) and thiobarbituric acid reactive substances (TBARS) values. In terms of prediction with leave-one-out cross-validation (LOOCV), PLS provided a higher accuracy for TBARS ($R^{2}$ = 0.801), while PV was lower ($R^{2}$ = 0.469). However, the auto-prediction showed good agreement among the observed and predicted values in both PV ($R^{2}$ = 0.802) and TBARS ($R^{2}$ = 0.932). Based on the variable importance of projection (VIP) scores, the ATR-FTIR peaks as 3000–2825, 2154–2150, 1780–1712, 1487–2483, 1186–1126, 1107–1097, and 1012–949 cm^−1^ were identified to be the most related to PV and TBARS on green coffee beans shelf life. ATR-FITR showed potential as a fast and accurate technique to evaluate the oxidation reaction that related to the loss of coffee quality during storage.

## 1. Introduction

The global production and consumption of coffee are supported by a comprehensive market analysis, appreciated for its rich flavor, aroma, and stimulating properties [1]. Arabica has fallen 7.1% to 94 million bags, while Robusta is set to rise 5.1% to 73 million bags [2]. Several factors influence the final quality of coffee, including climate change [3], post-harvest [4], coffee processing [5,6,7], and storage [8,9,10], which negatively impact coffee quality assessment and the sensory characteristics of coffee, as well as the obtaining of optimal and market prices [11]. Additionally, maintaining the quality of coffee throughout its shelf life presents a significant challenge for Thai coffee producers, distributors, and consumers.

As coffee ages, it undergoes chemical changes that degrade its sensory attributes, leading to a loss flavor, aroma, and overall appeal [8,9,10,11]. Also, the quality of the coffee could change while it is being stored, causing mold to grow and harmful compounds to develop. This could affect consumer health, manufacturing costs, and safety, depending on the beans’ physicochemical properties, oxidation reactions, storage time, and environmental conditions [11,12,13]. Thus, moisture content, density, lipid oxidation, and other chemical changes affect coffee odor, flavor, aroma, and overall sensory attributes [14,15,16]. To maintain the quality during storage, GrainPro^®^ was applied, and it presented better results than other packaging [16,17]. However, coffee still undergoes oxidation when exposed to air, light, and heat, resulting in the formation of volatile compounds such as aldehydes and ketones that are related to off flavors and rancid aromas [10,18,19], as well as the characteristic rich and nuanced flavor of coffee deteriorating, resulting in a flat taste [15]. Furthermore, accelerated storage tests that measure peroxide (PV) and thiobarbituric acid reaction substances (TBARS) values provided insights into the extent of lipid oxidation. Monitoring these values helps to assess the coffee’s susceptibility to oxidative degradation and provides a quantitative means to evaluate shelf life [15,20]. A kinetic model helps to predict shelf life using accelerated storage tests. Using an Arrhenius model shows how temperature influences degradation processes, enabling accurate predictions of shelf life under different conditions [15,20,21]. Thus, understanding the factors influencing coffee deterioration and accurately assessing its shelf life are crucial for ensuring product quality and consumer satisfaction. The applied shelf life under accelerated storage could be subjective, time-consuming, and impractical for large-scale productions.

The adequate packaging, storage, and utilization of green coffee beans and roasted coffee beans are essential while applying non-destructive methodologies for inspecting, testing, and assessing the coffee characteristics without causing damage to the original parts [22,23]. The FTIR spectrum can detect primary vibrations within the mid-infrared range, specifically between 4000 and 400 cm^−1^. These vibrations arise when molecules absorb overtones and combinations of fundamental vibrational bands [24,25,26]. Fourier transform infrared spectroscopy (FTIR) is a non-destructive technique that is used to assess the physical and chemical attributes, reactions, aroma compounds, sensory quality, and overall quality of green and roasted coffee beans [27,28]. According to Barrios-Rodriguez et al. [27], the ATR-FTIR method could be used as an extra way to tell the difference between, and describe the taste outcomes of, different coffee processing (natural, semi-dry, and washed processes) and roasting conditions (medium and dark roasted). Additionally, ATR-FTIR has presented a valuable formation of the chemical composition of green and roasted coffee, e.g., water activity, moisture content, caffeine, lipid, chlorogenic acid (CGA), carbohydrate, trigonelline, and other compounds [24,29]; the classification of the coffee variety [30]; the fermentation technique [31]; the geographical location [32,33]; defects [34,35]; adulterants [36]; the determination of antioxidants [37,38]; the quality of specialty coffee [39]; and sensory characteristics [40,41]. However, there is limited reporting on the use of ATR-FTIR on coffee shelf life at the present. By employing ATR-FTIR, researchers and coffee producers can gain valuable insights into the chemical changes occurring in coffee over time, helping them determine its shelf life and optimize storage conditions to maintain quality. The inherent chemical fingerprint of coffee, ATR-FTIR allows for the rapid and non-destructive analysis of key components involved in coffee degradation.

In order to establish a direct relationship between the spectral characteristics and the shelf life based on PV and TBARS values, multivariate analysis was applied. According to Tandee et al. [42], the calibration model was developed using partial least squares (PLS) regression. The predictive model PLS is effectively utilized in various applications, e.g., discriminating defects of coffee [43], classifying sensory attributes [40], and characterizing chemical compositions [44,45]. Thus, the efficacy of PLS regression with FTIR data could develop robust and accurate calibration models for predicting coffee shelf life from PV and TBARS values, ultimately enhancing quality control and product development processes in the coffee industry. Hence, the objective of this study was to investigate the shelf life of green Arabica coffee beans from both natural and honey processes based on lipid oxidation reactions by their shelf-life determination with ATR-FTIR under accelerated storage conditions.

## 2. Materials and Methods

### 2.1. Sample Preparation

**Figure 1:**
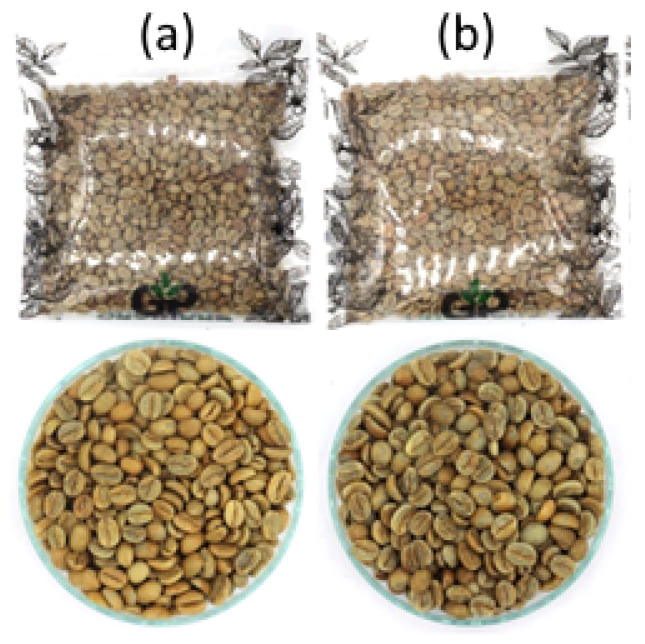
Packages for green coffee bean storage. GrainPro^®^ bags (**a**) natural process, and (**b**) honey process.

**Figure 2:**
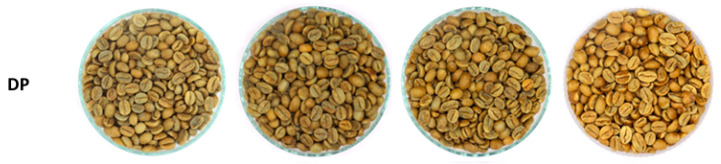

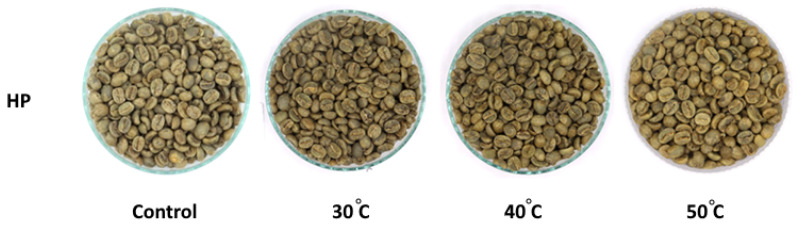
Green coffee bean storage in GrainPro^®^ bags after 20 days under accelerated storage condition.

The coffee cherries (*C. arabica* L. cv. Catimor) used in this study were obtained from Doi Thep Sadet, Chiang Mai, Thailand during the 2021/2022 coffee cherry harvesting season at an altitude of 1300 to 1400 m, latitude 18.95156 north and longitude 99.3484 east. The freshness of the coffee cherries was carefully observed and controlled throughout the processing stage. All unnecessary materials such as dust, dirt, leaves, twigs, and floating cherries were removed, and whole coffee cherries were dried to a moisture content (MC) of 10 ± 1% to produce the dry process (DP) samples. After de-pulping to get rid of the outer peel, the honey process (HP) samples were immediately dried until the MC reached 10 ± 1%. Then, 250 g of green coffee bean samples were placed into GrainPro^®^ bags (GrainPro Inc., Washington, DC, USA) as shown in Figure 1. GrainPro^®^ bags have the following specifications: an air permeability rating of 0.538 ± 0.04, a thickness of 0.124 ± 0.00 mm, and a surface area of 0.0445 m${\;\;}^{2}$. The measurement of air permeability was carried out using an approved air permeability tester (FX 3300 Lab Air IV, Textest Instruments, Schwerzenbach, Switzerland) in compliance with the guidelines provided in ASTM D737-04. The parcels were divided into 20 × 20 cm${\;\;}^{2}$ pieces, and the results were recorded in units of L/m${\;\;}^{2}$. The thickness of the packages was measured using a thickness gauge (SMD-565J, Teclock, Nagano, Japan). The coffee bags were stored in a Constant Climate Chamber (HPP750, Memmert GmbH, Schwabach, Germany) with the temperature controlled at 30 °C, 40 °C, and 50 °C at 50% RH. Coffee samples were removed from the chamber every 5 days (0, 5, 10, 15, and 20 days). The samples were then packed into polyethylene vacuum-sealed packets and kept at −80 °C in an ultra-low temperature freezer (MDF-193, SANYO, Osaka, Japan) before quality determination. The day 0 samples, which served as the control, were created without packaging. Figure 2 shows the appearance of the green coffee beans at day 20.

### 2.2. Moisture Content (MC)

Moisture content (MC) was measured by drying the coffee beans in a hot air oven at 70 ± 1 °C for 16.0 ± 0.5 h to achieve constant weight according to the AOAC method 979.12 [46] and calculated using Equation (1). The samples were taken in three replicates and expressed as percentages on a wet basis (%w.b.):

(1) $$\%\mathrm{Moisture}=\frac{\mathrm{Weight}\;\mathrm{loss}\;\mathrm{on}\;\mathrm{drying}\;\left(g\right)}{\mathrm{Weight}\;\mathrm{of}\;\mathrm{sample}\;\left(g\right)}\times 100$$

### 2.3. Water Activity ($a_{w}$)

The water activity ($a_{w}$) of the ground green coffee bean samples was determined using a water activity meter (Aqua Lab, Decagon, WA, USA) at 25 °C with auto-analysis. $a_{w}$ values were recorded as the mean of the three replicates [47].

### 2.4. Coffee Oil Extraction

Coffee oil samples were prepared using solvent extraction as described in previous studies [15,16]. Petroleum ether (400 mL) in a 500 mL Erlenmeyer flask was used to extract a 50 g sample of green coffee powder, utilizing an ultrasonic water bath (SS 304 Automatic Ultrasonic Water Bath, ASK-06A LAB Instruments, Hyderabad, India) at room temperature up to 25 °C for 30 min. The petroleum ether was then evaporated using a vacuum rotary evaporator after the samples had been filtered using Whatman Paper No. 4. A 50 mL sample vial constructed of amber borosilicate glass was then used to transfer the extracted oil from the collected oil. The vial had a rubber cap to create a tight polytetrafluoroethylene seal. The oil was kept at 2 °C until subjected to additional examination.

### 2.5. Lipid Oxidation Determination

#### 2.5.1. Peroxide Value (PV)

The PV was measured following the AOAC Official Method 965.33 [47]. One gram of oil sample (S) and a blank (B) were first prepared in two 250 mL Erlenmeyer flasks. Then, 30 mL of a mixed solvent of chloroform–acetic acid (2:3 *v*/*v*) was added. Next, each flask received 0.5 mL of saturated KI solution and was shaken and left to stand in the dark for 1 min. The reaction was stopped by adding 30 mL of distilled water to the combined samples. The mixture was then titrated with 0.002 M sodium thiosulfate until the blue tint disappeared. The outcome was recorded in milliequivalents of peroxide per kilogram of oil (meq/kg of oil) using Equation (2):

(2) $$\mathrm{PV}(\mathrm{meq}/\mathrm{kg}\;\mathrm{samples})=2\times \frac{\mathrm{Sample}\;-\;\mathrm{Blank}\;\left(\mathrm{mL}\right)}{\mathrm{Weight}\;\mathrm{of}\;\mathrm{oil}\;\left(g\right)}$$

#### 2.5.2. Measurement of Thiobarbituric Acid Reactive Substances (TBARS)

The measurement of TBARS followed Rendon et al. [4]. First, 4 mL of 1% (*w*/*v*) trichloroacetic acid (TCA) containing 0.08 g of polyvinylpolypyrrolidone (PVPP) and 0.2 g of the ground coffee sample were combined. The mixture was then continuously homogenized for 30 min before centrifuging for 10 min at 7 °C at 20,000 rpm. A 20% TCA (*w*/*v*) solution was added, and the supernatant contained 0.5 mL of 0.5% (*w*/*v*) thiobarbituric acid. The reaction mixture was placed in a water bath for 30 min before cooling and centrifuging at 10,000 rpm, 10 °C for 10 min. Quantification was performed by a spectrophotometer (Agilent Technologies, Santa Clara, CA, USA) at 532 and 600 nm using an extinction coefficient of 155 mM^−1^ cm^−1^, with results expressed as nmol of MDA/g of sample (d.w.).

### 2.6. Shelf-Life Prediction

Kinetic models were utilized to calculate the shelf-life prediction of green coffee beans [15,20], and integrated using the Arrhenius equation. This related the rate of time to three specific temperatures (30 °C, 40 °C, and 50 °C) under accelerated storage conditions with 50% RH. Equations (3)–(6) are presented below. The kinetic parameters were estimated using Microsoft Excel (version 2405):

(3) $$\mathrm{Zero}-\mathrm{order}\;\mathrm{model}:\;PV=k_{0}t+PV_{0}$$

(4) $$\mathrm{First}-\mathrm{order}\;\mathrm{model}:\;PV=kt+ln(PV_{0})$$

(5) $$\mathrm{Arrhenius}\;\mathrm{equation}:\;ln\left(k\right)=-Ea/RT+ln\left(k_{0}\right)$$

(6) $$\mathrm{Shelf}-\mathrm{life}\;\mathrm{prediction}:\;SL=\left[ln\left(PV\right)-ln\left(PV_{0}\right)\right]/\left[k_{0}e\left(-Ea/RT\right)\right]$$

where $k_{0}$ and *k* are the reaction rate constants. *PV* and $PV_{0}$ are the $PV_{s}$ at storage time. *t* is the initial value. $k_{0}$ is a pre-exponential factor, and *Ea* is the activation energy (J mol^−1^). T is the absolute temperature, *R* is the molar gas content (8.3144 J K^−2^ mol^−2^), and *SL* is the shelf-life prediction.

### 2.7. FTIR Spectroscopy Analysis

A Fourier Transform Infrared (ATR-FTIR) spectrophotometer (Cary 630, Agilent Technologies, Santa Clara, CA, USA) [24,27] was used to conduct the analysis. The coffee samples were ground using a grinder to achieve a particle size of 200 ± 25 μm. The spectrophotometer was outfitted with a deuterated L-alanine doped triglycine sulfate (DLATGS) detector and a diamond attenuated total reflectance (ATR) sample attachment. All measurements were conducted within the 4000 to 400 cm^−1^ spectral region at a resolution of 4 cm^−1^ and 16 scans in a dry atmospheric environment at room temperature (20 ± 0.5 °C).

### 2.8. Statistical Analysis

The results of MC, $a_{w}$, PV, and TBARS were recorded as mean values of three replicates with standard deviations (*n* = 3). Analysis of variance (ANOVA) was carried out using SPSS statistical software (version 20, SPSS Inc., Chicago, IL, USA). Tukey’s test (*p* ≤ 0.05) was used to compare the mean data and identify significant variations between the treatments. MATLAB^®^ (version 7.9.2009, MathWorks, Natick, MA, USA) was used to perform partial least squares (PLS) regression and principal component analysis (PCA) using ATR-FTIR data along with other parameters, e.g., MC, $a_{w}$, PV, and TBARS, and shelf life.

## 3. Results and Discussion

### 3.1. Moisture Content and Water Activity Parameter of Green Coffee Beans

**Figure 3:**
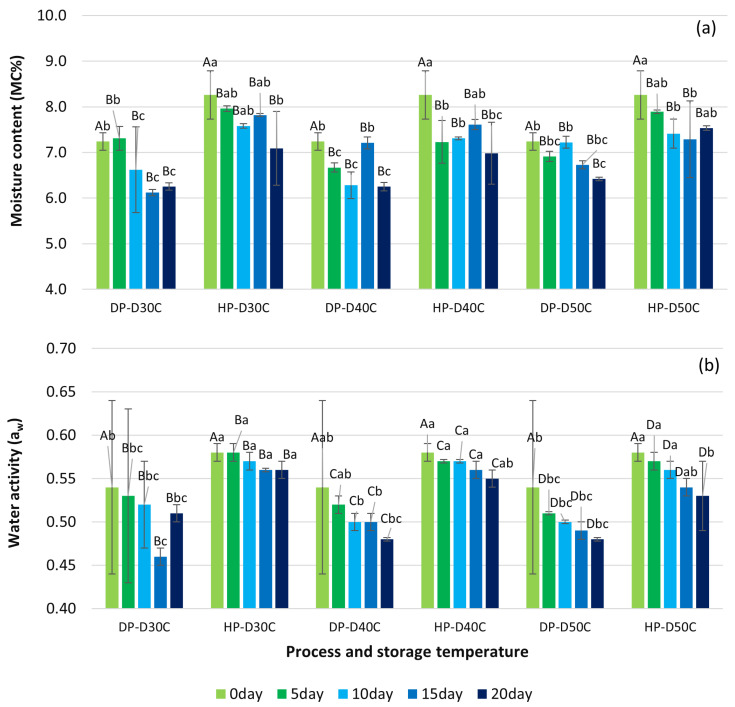
Effect of storage conditions on changes in (**a**) moisture contents and (**b**) water activity during accelerated storage conditions. Different capital letters indicate significant differences in temperature at *p* < 0.05; different lowercase letters indicate significant differences in storage time at *p* < 0.05. DP-D30C: natural process storage at 30 °C; HP-D30C: honey process storage at 30 °C; DP-D40C: natural process storage at 40 °C; HP-D40C: honey process storage at 40 °C; DP-D50C: natural process storage at 50 °C; HP-D50C: honey process storage at 50 °C.

Under accelerated storage in GrainPro^®^ bags, the moisture content (MC) and water activity ($a_{w}$) values of green coffee beans from the natural process (DP) and honey process (HP) are shown in Figure 3a,b. The MC and $a_{w}$ values of green coffee beans from different processing conditions significantly reduced (*p* < 0.05) after 20 days of accelerated storage in GrainPro^®^ bags at 30 °C, 40 °C, and 50 °C. Achata et al. [48] stated the most important attributes when assessing the quality and stability of dried food shelf life as MC and $a_{w}$. The MC of DP and HP coffee beans ranged from 7.31 ± 0.26 to 6.12 ± 0.07 and from 8.26 ± 0.53 to 7.09 ± 0.81, respectively, after 20 days of storage. The optimal MC of a green coffee bean is between 9% and 12%; hence, the sample was lower than the normal MC ranges. Green coffee beans with low MC produce the unsatisfactory results of an unpleasant aroma and poor-quality beans [16]. Under storage, MC is impacted by chemical interactions and oxidative deterioration in the packaging, leading to rancid aromas, quality loss, and secondary oxidation [16,49].

The $a_{w}$ reductions in DP and HP coffee beans ranged from 0.54 ± 0.04 to 0.46 ± 0.01 and 0.58 ± 0.01 to 0.53 ± 0.04, respectively. By contrast to the HP procedure, DP coffee beans gave the most significant reduction in $a_{w}$ during a 20-day storage period. The storage of samples at a lower temperature of 30 °C resulted in a higher $a_{w}$ content compared to storage at 40 °C and 50 °C. This finding concurred with Michalak et al. [50], who also observed elevated $a_{w}$ levels at a significantly lower temperature of 25 °C. Orfanou et al. [51] found that shelf life was significantly influenced by storage temperature and $a_{w}$. Values of $a_{w}$ below 0.52 significantly affected the sensory attributes of coffee including aroma intensity, fragrance quality, aftertaste, and the formation of off flavors [51]. The shelf life was around 20 days when the $a_{w}$ value was below 0.36 [21]. Manzocco and Nicoli [52] presented empirical findings indicating that the parameter $a_{w}$ exhibited temperature dependency, resulting in alterations to both the apparent activation energy and the frequency factor within the framework of the modified Arrhenius equation. When the $a_{w}$ value is between 0.52 and 0.8, an Arrhenius-type correlation exists between the rate of H3O^+^ generation and the temperature [52].

### 3.2. Oxidation Reactions of Green Coffee Beans

**Figure 4:**
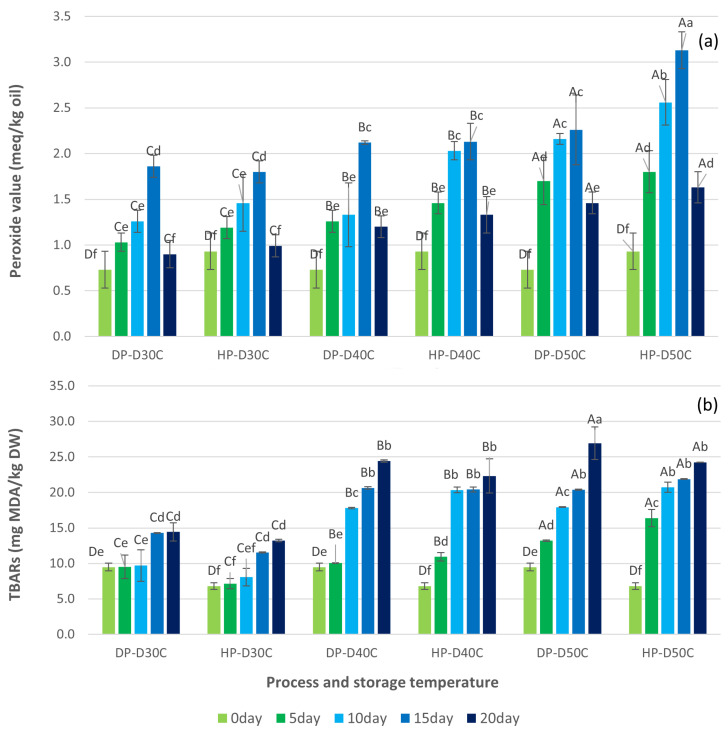
Changes in the oxidation reactions of green coffee beans during the natural and honey processes; (**a**) peroxide value, and (**b**) TBARS. Different capital letters indicate significant differences in temperature at *p* < 0.05; different lowercase letters indicate significant differences in storage time at *p* < 0.05. DP-D30C: natural process storage at 30 °C; HP-D30C: honey process storage at 30 °C; DP-D40C: natural process storage at 40 °C; HP-D40C: honey process storage at 40 °C; DP-D50C: natural process storage at 50 °C; HP-D50C: honey process storage at 50 °C.

Shelf life, volatile loss, physical collapse, loss of pleasant fragrance components, and off-flavors are all impacted by the rate of coffee oxidation reactions [20]. The two most significant lipid oxidation statistics were PV and TBARS [15,20]. Figure 4 shows the PV and TBARS values for DP and HP. Aung Moon et al. [16] also reported that PV and TBARS values of the washed process changed with increasing storage temperature and storage time. The PV developed as a primary reaction, while TBARS presented as secondary oxidation. Parvathy et al. [53] recorded the main sources of lipid oxidation as PV and TBARS, giving unfavorable alterations to flavor, aroma, texture, color, and nutritional value. The peroxide value (PV) concentration of DP and HP varied within the ranges of 0.73 ± 0.20 to 2.26 ± 0.06 and 0.93 ± 0.20 to 3.13 ± 0.20 meq/kg oil, respectively. The PV values obtained from DP and HP after 20 days of accelerated storage were below 3.13 meq/kg oil. Anese et al. [21] also reported PV values below 2 meq/kg oil, while Yoon et al. [54] reported PV values below 3 meq/kg in a milk beverage mixed with coffee extract. The PV value increased until 15 days before physical changes occurred, causing a disagreeable scent and off-flavor. The PV is a major product generated in oil by auto-oxidation processes with an increase in hydroperoxide reported by Cong et al. [15]. PV production occurs exponentially, with accumulation until subsequent reactions produce breakdown products such as aldehydes [15,55]. PV increases due to the introduction of highly reactive radical species into the product, which promotes oxidation [55]. This may lead to a reduction in the quality of the coffee and its ability to be stored for extended periods.

The highest PV content was found in HP, followed by DP. HP reacted with mucilage-dried coffee on both skin-dried and parchment-dried coffee, producing yellow, red, and black honey coffee [5,56]. Microorganisms devoured sugars and other substances on the mucilage section and then broke them down into byproducts that were absorbed into the cellular structure of the green coffee bean, leading to a more yellow-brown bean than the washing procedure [57,58]. The TBARS contents of DP and HP ranged from 9.48 ± 0.55 to 26.92 ± 2.28 and 6.81 ± 0.46 to 24.25 ± 0.03 mg MDA/kg DW, respectively. The value of TBARS increased during accelerated storage, consistent with the outcome described by Rendon et al. [4]. Thiobarbituric acid (TBA) and malonaldehyde (MDA) were formed during the final stage of lipid oxidation by the breakdown of hydroperoxides obtained from fatty acids with three or more double bonds and reacted in the TBARS test [4,15]. Following 20 days of accelerated storage, the TBARS values of DP and HP were comparatively higher than those reported by Aung Moon et al. [16] for washed processing. DP and HP may develop a more undesirable odor and a greener appearance. As a result, the fungi and mold that produced the auto-oxidation, photo-oxidation, and enzymatic oxidation reactions had a higher impact on HP and DP during storage. After three months of storage, Borem et al. [9] noted that DP green coffee beans packed in permeable paper bags had sensory quality damage, with a cardboard or old crop flavor. However, HP changes were noted during the ninth month of storage. Natural coffee loses its sensory qualities faster than pulped natural coffee or honey [9]. TBARS was consistent with coffee bean respiration being interrupted during storage at temperatures lower than 40 °C and 50 °C. TBARS are lipid oxidation byproducts that combine with proteins to create polymers [4]. TBARS levels are more sensitive to ketones, esters, pyridines, and other chemicals [15].

### 3.3. Shelf Life of Green Coffee Beans

A kinetic model and Arrhenius equations were used to investigate the shelf life of green coffee beans. This approach has been used to predict the shelf life of green coffee beans [15], coffee brew [59], and capsule packaging [60]. Table 1 shows the shelf life of green coffee beans calculated based on the different lipid oxidation parameters, e.g., PV and TBARS. The shelf life of DP at 30 °C, 40 °C, and 50 °C was 35.57, 25.64, and 21.10 days, with HP 51.50, 33.88, and 15.26 days, respectively. The secondary oxidation of TBARS, the shelf life of DP was 10.1, 4.25, and 3.82 days, and HP was 8.84, 4.19, and 3.96 days at 30 °C, 40 °C, and 50 °C, respectively. Green coffee beans stored at 30 °C had a longer shelf life than those stored at 40 °C and 50 °C, with HP processing showing a longer shelf life than DP processing at 30 °C. HP drying with parchment and mucilage can dehydrate moisture content and reduce water activity faster during drying and storage, thereby impacting the transition to a whiter bean, the shade size, and the yellowish color [8]. HP and DP had lower shelf life at high-temperature storage of 50 °C. By contrast, the $a_{w}$ impact on the kinetics of shelf-life deterioration was attributed to the involvement of non-enzymatic browning [61].

### 3.4. ATR-FTIR Spectra of Green Coffee Beans

**Figure 5:**
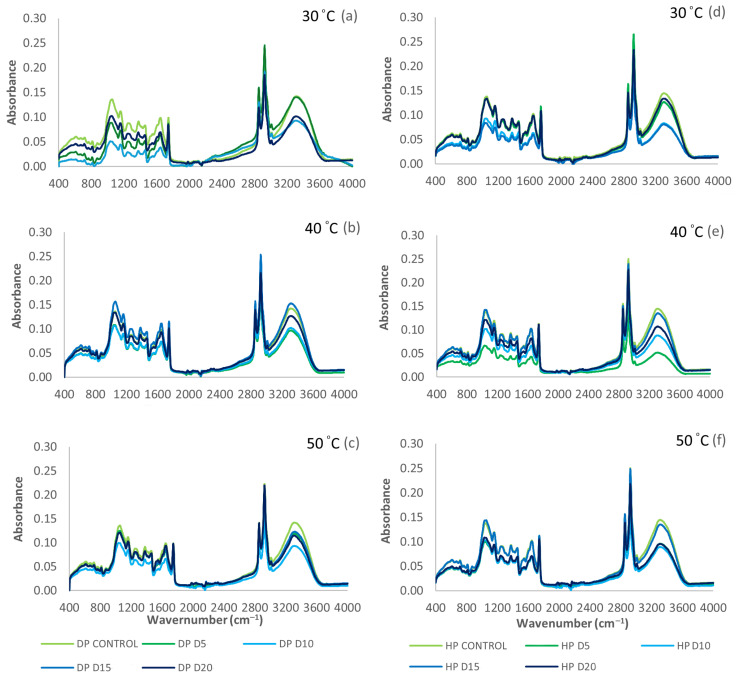
ATR-FTIR spectra of DP green coffee bean storage at (**a**) 30 °C, (**b**) 40 °C, and (**c**) 50 °C and HP storage at (**d**) 30 °C, (**e**) 40 °C, and (**f**) 50 °C.

The FTIR technique was used to explore the physicochemical structures of DP and HP, as well as their mixtures at different temperatures (30 °C, 40 °C, and 50 °C) and storage times (0, 5, 10, 15, and 20 days). The absorbance region of green coffee beans ranged from 4000 to 400 cm^−1^. The same peak structure line-up was shown in the infrared spectra of DP (Figure 5a–c), and HP (Figure 5d–f) under varied accelerated storage conditions. The spectrogram showed the main regions of 12 absorbance peaks at wavenumbers 3313, 3010, 2854, 1745, 1645, 1458, 1377, 1248, 1157, 1049, and 715 cm^−1^. Fagan and O’Donnell [62] divided the wavenumbers into two groups: a functional group, 4000 to 1450 cm^−1^, and a fingerprint group, 1450 to 400 cm^−1^.

The absorbance peak at 3313 cm^−1^ was attributed to the stretching of O-H bonds in relation to $a_{w}$ and MC [24,62]. This peak was also connected with the stretching vibration of O-H bonds in hydroperoxides 3444 cm^−1^ [37], as well as the stretching of O-H bonds in alcohols and water (3280 cm^−1^) by Tsiaka et al. [63]. After a 20-day storage period, the coffee beans exhibited decreased MC and $a_{w}$ values. HP showed the smallest loss, followed by DP. DP involved longer sun drying than the other process, which caused a decrease in O-H stretching associated with water molecules, with reduced MC and $a_{w}$ [24,29]. During drying, fermentation occurs, leading to the creation of hydroperoxides and causing a decrease intensity of the O-H stretching peak at 3444 cm^−1^ [37]. Using a regulated fermentation process (DP technique) reduced the production of alcohols, thereby impacting O-H stretching 3280 cm^−1^ [37].

The wavenumber peaks observed within the range of 3010 cm^−1^ were associated with the symmetric vibration of C-H stretching in cis double bonds 3009 cm^−1^ as reported by Raba et al. [37]. These peaks might also be attributed to C-H stretching in aromatic rings (3130– 3010 cm^−1^) as described by Tsiaka et al. [63]. The efficacy of the DP and HP methods diminished over 20 days of accelerated storage. The peaks at 2925 and 2854 cm^−1^ were attributed to the antisymmetric stretching of CH_2_ and CH_3_ groups in lipids as reported in previous studies [27,62,63]. Raba et al. [37] provided a description of the asymmetric and symmetric stretching vibrations of the C-H bond inside the aliphatic CH_2_ group of the fatty acid backbone and also discussed the C-H symmetrical stretching of methyl groups ( 2850 cm^−1^) as reported by Wang and Lim [29]. Conversely, the vibration of the C-H bonds in both caffeine and lipid molecules may exert an influence [24,39]. When comparing the processing methods, DP at 50 °C resulted in a significantly decreased peak between 2925 and 2854 cm^−1^. No substantial alteration was observed in the chemical characteristics of HP. Extended periods of drying can cause an elevation in lipid oxidation and degradation, leading to a reduction in the intensity of CH_2_ and CH_3_ stretching vibrations, hence accelerating the observed alterations and reductions. The utilization of HP techniques may result in reduced drying durations, with less significant alterations in the aforementioned peaks. The potential impact of temperature on CH_2_ and CH_3_ stretching vibrations may be mitigated to a certain degree by the stability of lipids throughout these processes [62,63]. The potential influence of coffee and its interaction with lipids on these peaks could result in variances in the reported decreases [27].

The absorbance peak at 1745 cm^−1^ corresponded to the stretching of the C=O bond in lipids [62], the stretching of the C=O bond in aliphatic esters [63], and the stretching vibration of ester carbonyl functional groups in triglycerides (O-C=O) [37], as well as in chlorogenic acids (CGAs) and caffeine [28]. The peak at 1645 cm^−1^ was associated with the C=C stretching vibration in cis-olefins (cis RHC=CHR) [61], as well as in caffeine ( 1650 cm^−1^) and trigonelline (1600–1300 cm^−1^) [24]. The HP samples demonstrated greater intensity at wavenumbers 1745 and 1645 cm^−1^ in comparison to the DP samples. The higher peak seen in the HP samples indicated the enhanced preservation and accessibility of vibrations, highlighting the capacity to facilitate beneficial chemical modifications in the molecules under investigation, specifically in relation to the conformation and interactions of ester carbonyl functional groups.

Within the fingerprint regions, distinct absorbance peaks at specific wavenumbers 1458, 1377, 1248, 1157, 1049, and 715 cm^−1^ were related to chlorogenic acids (1450–1150 cm^−1^), C-H scissoring bend of CH_2_ (1485–1445 cm^−1^), OH bend in organic acids (1381–1376 cm^−1^), saccharose ( 1237 cm^−1^), C-N stretch (1241–1218 cm^−1^), C-O stretch in organic acids (1161–1153 cm^−1^), arabinogalactans (1065–1020 cm^−1^), cellulose or quinic acid (1082–1033 cm^−1^), carbohydrate (150– 700 cm^−1^), overlapping of aliphatic CH_2_ rocking vibration, and the out-of-plane vibration of cis-disubstituted olefins (722 cm^−1^), respectively [24,27,37,62,63]. Consequently, the DP peak exhibited the least amount of content at 30 °C in the fingerprint group, whereas HP displayed relatively lower content compared to 40 and 50 °C. At a lower temperature of 30 °C, certain interactions decreased favorability, leading to a decrease in the concentration of molecules linked to specific absorbance peaks in DP. However, when exposed to temperatures ranging from 40 and 50 °C, these interactions exhibited a greater propensity toward green coffee beans, resulting in an augmented concentration of chemicals in DP. The impact of temperature fluctuations on the performance of HP diminished, depending on specific processing techniques or inherent chemical stability. Thus, the ATR-FTIR absorbance peak area could be continuously affected by physicochemical composition, metabolic features, lipid oxidation, and the presence of important compounds, e.g., carbohydrates, proteins, lipids, caffeine, CGAs, and trigonelline, corresponding to the functional group and fingerprint group peaks.

### 3.5. Multivariate Analysis of Coffee Shelf Life Under Accelerated Storage

**Figure 6:**
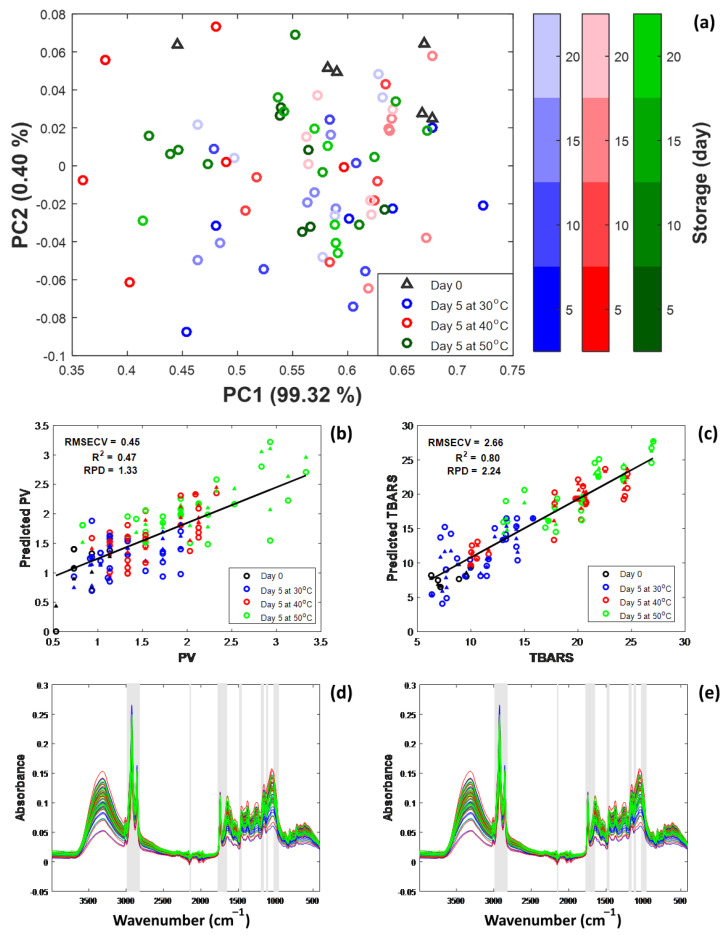
PCA score plot of PC1 against PC2 with the oxidative parameters during accelerated storage (**a**). The correlation graphs present the expected and predicted PV (**b**) and TBARS (**c**) values (circle symbols present the LOOVC, while triangle symbol presents the Auto-Prediction). The overlap VIP scores (highlighted zone means VIP ≥ 1) with FTIR spectrum of PV (**d**) and TBARS (**e**).

The score plot of the lipid oxidation is shown in Figure 6a, representing 99.72% of the total variation. A difference between the storage duration and temperature was observed. This meant that PCA could be used to find the basic structure in the ATR-FTIR data of 1869 peaks, with a wavenumber range of 401 to 4000 cm^−1^ and a PV and TBARS of variation in the dataset at 26 samples across three replications (78 samples). The control samples clustered on the top right of the plane showed the similarity of the samples prior to storage. When the accelerated storage proceeded, the clustering of data points at the center of the plane showed an increase in oxidative reactions, resulting in an increase in PV and TBARS, while the shelf life was lower.

Based on the experimental data, PLS models were established; the performance values and PLS parameters are shown in Table 2. In both cases, the $R^{2}$ values were greater in the auto-prediction of 0.802 for PV and 0.932 for TBARS. This showed a good explanation among the prediction results and the input variables. But when leave-one-out cross-validation (LOOCV) was used with SNV and 1^st^ derivative pre-processing, the PV had a lower $R^{2}$ of 0.469, while the TBARS had a higher $R^{2}$ of 0.801. The root mean square error of cross validation (RMSECV) was 0.449 and 2.658 for PV and TBARS, respectively. In this case, the PLS results showed that ATR-FTIR was better at measuring the oxidation reactions of TBARS than PV. This might be due to the characteristics of the primary oxidation of PV that are lower when the storage time increases, while TBARS gradually increases (Figure 4). Figure 6b,c show the plots obtained between the observed and predicted values for the models of the oxidation reactions of PV and TBARs. It was evident that the impact of a high storage temperature (50 and 40 °C) provided a high content of predicted PV and TBARS when compared to control and storage in lower conditions with a shorter storage time. As a result, the study used the PLS model, which it achieved by locating and removing outliers. These techniques were effective in predicting and establishing a relationship between ATR-FTIR data and oxidation reactions on shelf-life parameters.

The partial least squares–variables important of projection (PLS-VIP) plots for PV and TBARS are shown in Figure 6d,e. It is crucial to acknowledge that the PLS-VIP indicates the significance of the parameters in the context of the oxidation data prediction. The size of VIP parameters can be used to determine how significant the variables are or how influential they are for the prediction model [42]. Based on the PLS-VIP, ATR-FTIR peaks of 3000–2825, 2154–2150, 1780–1712, 1487–2483, 1186–1126, 1107–1097, and 1012– 949 cm^−1^ were identified to be strongly influential for the model prediction of PV and TBARS. Additionally, the observations were reached at 3000–2854 and 1745 cm^−1^ peaks, which decided the impact of the moisture content and water activity during the accelerated storage, and 2925 to 2854 cm^−1^ was considered to be the C-H asymmetric and symmetric stretching of CH_2_ and CH_3_ in the lipids, caffeine, aliphatic compounds, and fatty acids [61,62]. On the other hand, these aspects contributed to organic molecules such as lactone, aliphatic esters, aldehydes, ketones, aliphatic acids, and carboxylic acid, which played a role in extending the longevity of unroasted coffee beans [29,39]. In the past, researchers have looked into how the C=O bond in compounds like caffeine, trigonelline, and chlorogenic acid affects their ATR-FTIR absorbance spectra, finding a peak between 1645 and 1458 cm^−1^ [27,39,64]. The absorbance peak at 1377, 1157, and 1049 cm^−1^ could be related to the vibrational modes of O-H bonds in organic acids, the presence of C-O ester groups, the stretching of C-N bonds in organic acids, and the composition of the cellulose ester group in green coffee beans. These vibrational modes affect the production of quinic acid [27,29,63]. Thus, in turn, this has implications for the extended preservation of the beans. As a result, the identified ATR-FTIR peaks, along with other characteristics, give us a lot of information about the chemical changes that happen during accelerated storage. Subsequent research endeavors should focus on the utilization of these discoveries to optimize coffee storage and improve methods related to quality management.

## 4. Conclusions

The shelf life of green coffee beans stored in GrainPro^®^ bags was investigated by assessing the levels of lipid oxidation, namely, peroxide and TBARS. At a storage temperature of 30 °C, the shelf life of the honey coffee process was longer than the natural process. Long periods of green coffee bean storage were conducted at low temperatures. ATR-FTIR spectroscopy and lipid oxidation parameters were utilized for the assessment and management of green coffee bean quality. Green coffee beans with a longer shelf life exhibited a correlation with a lower temperature of 30 °C compared with 40 °C and 50 °C. According to PLS, the models showed acceptable prediction results for TBARS on green coffee beans. Moreover, the PLS-VIP revealed that the important wavenumber ranges of 3000–2825, 2154–2150, 1780–1712, 1487–2483, 1186–1126, 1107–1097 and 1012– 949 cm^−1^ are considered related to the lipid oxidations. Thus, the correlation between oxidation reactions and ATR-FTIR analysis in green coffee beans provided the evidence demonstrating the influence of processing treatments on the shelf life of coffee beans, which could be used to monitor the quality of coffee.

## Figures and Tables

**Table 1:** Shelf life of green coffee beans with different processing methods and storage temperatures.

Temperature (°C)	Process	PV		TBARS	
		$R^{2}$	Shelf Life (Days)	$R^{2}$	Shelf Life (Days)
30 °C	DP	0.95	35.57 ± 1.69 ^b^	0.99	10.01 ± 0.27 ^a^
	HP	0.89	51.50 ± 1.83 ^a^	0.99	8.84 ± 0.44 ^ab^
40 °C	DP	0.91	25.65 ± 1.67 ^c^	0.96	4.25 ± 0.10 ^c^
	HP	0.90	33.88 ± 2.00 ^b^	0.92	4.19 ± 0.23 ^c^
50 °C	DP	0.87	21.10 ± 3.71 ^cd^	0.98	3.82 ± 0.10 ^c^
	HP	0.99	15.26 ± 0.63 ^d^	0.93	3.96 ± 0.16 ^c^

Note: data are presented as mean ± SD. DP, natural process; HP, honey process. ^a–d^: Mean values with different superscript within the same columns indicate significance among different processing, temperature, and time at (*p* < 0.05).

**Table 2:** Relevant information and results obtained for the PLS models.

Response	Samples	Processing	LVs	LOOCV			Auto-Prediction		
				RMSECV	$R^{2}$	RPDCV	RMSE	$R^{2}$	RPD
PV	78	SNV and 1st derivative	10	0.449	0.469	1.332	0.264	0.802	2.260
TBARS	78	SNV and 1st derivative	10	2.658	0.801	2.237	1.546	0.932	3.846

Note: LVs—number of latent variables used, LOOCV—leave-one-out cross-validation, RMSE—root mean square error, RPD—residual predictive deviation, and cv—cross-validation.

## Data Availability

The original contributions presented in the study are included in the article, further inquiries can be directed to the corresponding author.
